# Differential neural network configuration during human path integration

**DOI:** 10.3389/fnhum.2014.00263

**Published:** 2014-04-29

**Authors:** Aiden E. G. F Arnold, Ford Burles, Signe Bray, Richard M. Levy, Giuseppe Iaria

**Affiliations:** ^1^NeuroLab, Department of Psychology, University of CalgaryCalgary, AB, Canada; ^2^Hotchkiss Brain Institute, University of CalgaryCalgary, AB, Canada; ^3^Departments of Radiology and Psychiatry, University of CalgaryCalgary, AB, Canada; ^4^Alberta Children’s Hospital Research Institute, University of CalgaryCalgary, AB, Canada; ^5^Faculty of Environmental Design, University of CalgaryCalgary, AB, Canada; ^6^Department of Clinical Neurosciences, University of CalgaryCalgary, AB, Canada

**Keywords:** navigation, spatial memory, partial least squares, dead reckoning, neural network

## Abstract

Path integration is a fundamental skill for navigation in both humans and animals. Despite recent advances in unraveling the neural basis of path integration in animal models, relatively little is known about how path integration operates at a neural level in humans. Previous attempts to characterize the neural mechanisms used by humans to visually path integrate have suggested a central role of the hippocampus in allowing accurate performance, broadly resembling results from animal data. However, in recent years both the central role of the hippocampus and the perspective that animals and humans share similar neural mechanisms for path integration has come into question. The present study uses a data driven analysis to investigate the neural systems engaged during visual path integration in humans, allowing for an unbiased estimate of neural activity across the entire brain. Our results suggest that humans employ common task control, attention and spatial working memory systems across a frontoparietal network during path integration. However, individuals differed in how these systems are configured into functional networks. High performing individuals were found to more broadly express spatial working memory systems in prefrontal cortex, while low performing individuals engaged an allocentric memory system based primarily in the medial occipito-temporal region. These findings suggest that visual path integration in humans over short distances can operate through a spatial working memory system engaging primarily the prefrontal cortex and that the differential configuration of memory systems recruited by task control networks may help explain individual biases in spatial learning strategies.

## INTRODUCTION

Humans and animals are able to spatially code their movement through environments using a combination of visual optic flow and self-motion cues (Mittelstaedt and Mittelstaedt, 1980; Etienne and Jeffery, 2004; Sargolini, 2006; Wolbers and Hegarty, 2010). This process is known as path integration, or dead reckoning, and is widely believed to be a fundamental navigational skill used to estimate self-location in an environment by tracking distances and directions from a given reference point. Theoretical models of path integration suggest that path information is encoded as a continuous movement vector that integrates visual, vestibular, and proprioceptive information to update both angular displacement and distance from a reference point within an environment (Loomis et al., 1999). These movement vectors allow for an efficient homing mechanism to quickly and accurately return to a particular location, but also, as some researchers have suggested, may provide the spatial scaffold upon which knowledge of landmark locations are associated to form allocentric-based spatial maps of an environment (Redish and Touretzky, 1997; Etienne and Jeffery, 2004; McNaughton et al., 2006; Moser et al., 2008; Müller and Wehner, 2010; Arnold et al., 2013).

Understanding the neural basis of path integration in animals has attracted considerable attention over the past decade (McNaughton et al., 2006; Sargolini, 2006; Jeewajee et al., 2008; Killian et al., 2012; Valerio and Taube, 2012), due to its potential to uncover the neural mechanisms of a fundamental navigation skill, as well as providing insight into principles of system-wide cortical dynamics and philosophical questions about how space is structured in the brain (O’Keefe and Nadel, 1978; Moser et al., 2008). This has been facilitated by the discovery of head direction cells (Taube et al., 1990) and grid cells (Fyhn, 2004; Hafting et al., 2005) that, together with place cells in the hippocampus (HC) (O’Keefe and Dostrovsky, 1971; O’Keefe and Nadel, 1978; O’Keefe and Burgess, 1996), are believed to provide the necessary neural computations for calculating an animal’s changing location using metric displacement and angular rotation (McNaughton et al., 2006; Sargolini, 2006; Moser et al., 2008). However, despite having numerous scientific implications, to date there has been little interest in understanding how path integration operates in humans at a neural level.

Wolbers et al. (2007) provided the first attempt to characterize the neural networks supporting human path integration by interrogating neural dynamics using functional magnetic resonance imaging (fMRI) while participants completed a virtual triangle completion task. In this study, the authors investigated whether variance in the blood oxygen level dependent (BOLD) signal measured during the outbound leg of a triangular path was related to within-subject consistency in pointing accuracy, and whether it could additionally explain between-subject differences in over- or underestimating the angular rotation needed to complete the triangular pathway. The study focused on investigating BOLD signal change within six regions of interest (ROIs) based on cortical and subcortical areas that had previously been shown to support path integration in animals (i.e., HC; entorhinal cortex; medial prefrontal cortex, mPFC; medial superior temporal cortex, MST; ventral intraparietal sulcus, VIP; retrosplenial cortex, RSC). Within subjects, the results revealed a five-voxel cluster within the right HC that had increased BOLD signal change correlating with more accurate pointing on a trial-by-trial basis. Between subjects, no differences in BOLD signal were found to correlate with a tendency to over or underestimate the angular rotation. However, increased BOLD signal change from a cluster within the bilateral mPFC and the right HC was found to positively correlate with higher response consistency; in addition, a bilateral increased BOLD signal change in MST was found to positively correlate with large systematic error scores, a measure of inability to encode self-motion (Fujita et al., 1993). From these results, the authors concluded that the HC plays a central role in integrating distance and direction signals processed elsewhere in the brain, while mPFC monitors and updates HC outputs coding a person’s current location. MST was hypothesized to assist in approximating self-motion from visual optic flow based on the known role of its homologue in the primate brain.

The selection of ROIs by Wolbers et al. (2007) was motivated by brain areas shown to support path integration in rodents. Recently, however, the central role of the HC underlying path integration has come into question in both animals (Sargolini, 2006; Moser et al., 2008) and humans (Shrager et al., 2008). A recent study by Kim et al. (2013) conducted parallel experiments on path integration in humans with medial temporal lobe lesions localized primarily in the HC and rats with hippocampal lesions. Patients were found to have preserved visual path integration ability over short distances (<50 s total travel time), while rats with hippocampal lesions showed behavioral deficits. This finding suggests that a working memory system located outside the medial temporal lobes may be used by humans to visually path integrate and raises the possibility that the neural mechanisms of path integration differs between humans and rats. However, neuropsychological research previous to Kim et al. (2013) have produced somewhat contrasting results on the of the HC in path integration. A study by Philbeck et al. (2004) investigated preserved path integration skills in a group of epileptic patients that had ~50% of the anterior portion of the left or right HC resected. The authors found that right, but not left, HC resection patients tended to overshoot linear paths that had been encoded visually but traversed blindfolded, thereby relying on vestibular and motion cues to estimate distance during movement. Similarly, a study by Worsley et al. (2001) found that right, but not left, HC resection patients were impaired on estimating a turn to return to a starting location after having been led blindfolded along two distances and a turn. In contrast to Kim et al. (2013), these studies suggest that the right HC is critical for non-visual path integration in humans.

Given the shifting perspective on the role of the HC in path integration and new evidence suggesting rats and humans may not engage the same neural system during this process, we sought to extend upon Wolbers et al. (2007) ROI-based approach and investigate path integration ability in humans using a data driven multivariate whole brain analysis. Specifically, we used partial least squares (PLS; McIntosh et al., 1996) to identify the spatial pattern of voxel activity across the brain that differentiated visual path integration from visual optic flow, as well as identifying the functional networks that correlated with performance on a path integration task. PLS is a multivariate technique similar to principle component analysis in which contrasts are typically not specified by the researcher. Rather, the PLS algorithm extracts latent variables (LVs) that maximally explain the covariance between voxel activity and experimental tasks while differentiating between each tasks. This allows for an unbiased estimate of differences in task related brain activity, in comparison to traditional ROI-based univariate approaches which are dependent on experimental hypotheses for region and contrast selection (McIntosh and Lobaugh, 2004). Additionally, PLS is able to assess differences in the functional connectivity of brain regions that relate to behavioral variability, allowing for an identification of the different neural networks that support task performance. As such, this statistical approach provides a comprehensive assessment of the distributed neural systems that underlie visual path integration, allowing for an extension upon previous ROI-based approaches that have characterized the functional specialization of specific brain regions supporting path integration.

## MATERIALS AND METHODS

### PARTICIPANTS

Fourteen healthy, right-handed volunteers (9 females, mean age 22.29 years, age range 18–36 years) with no psychiatric or neurological disorders, and normal or corrected-to-normal vision participated in the study. All participants provided written informed consent as approved by the Conjoint Health Research Ethics Board at the University of Calgary (CHREB 22848).

### PATH INTEGRATION TASK

**FIGURE 1:**
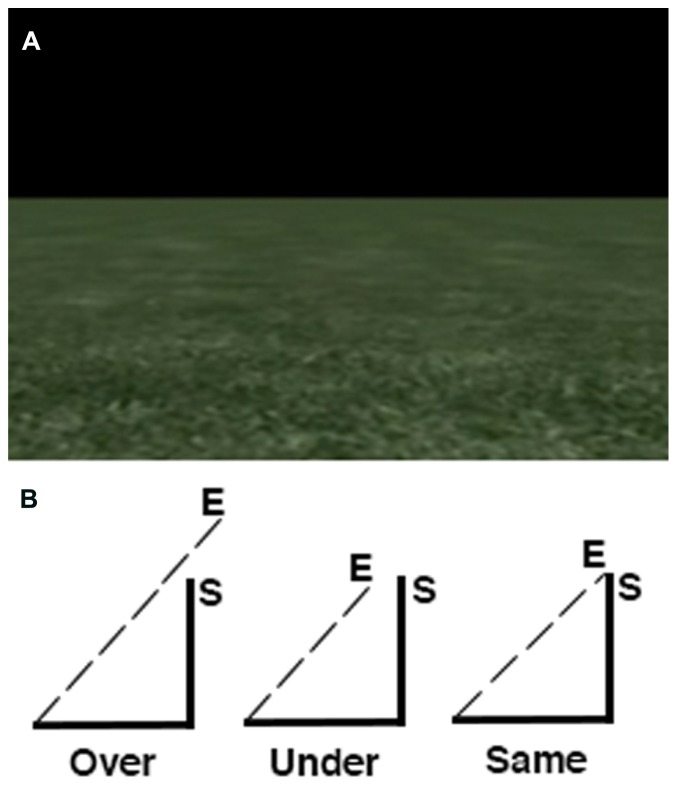
**Overview of the path integration task. (A)** Screen display depicting the textured ground with a horizon and black sky. **(B)** Distance estimations were always in relation to the last edge of the triangle.

The path integration task consisted of a single run with six experimental and six control trials. The order of trials was pseudo-randomized such that each run started and ended with a control trial. Experimental trials presented video clips of first person movement along the perimeter of an invisible right angle triangle through a virtual environment consisting of a textured floor and a horizon with a black sky (**Figure 1**). This environment was visually similar to the one developed by Wolbers et al. (2007). In the task, participants were provided with optic flow cues based on the moving texture of the floor, allowing for an estimation of distance being traversed within the environment. No other visual cues were present (e.g., landmarks or other salient environmental features). At the end of each video clip, participants were asked to indicate whether or not they believed the movement in the video ended at the same point it started. Of the six experimental trials, two trials ended before the starting point, two on the starting point and two after the starting point. The total path time for experimental trials ranged between 36 and 46 s. Two sets of triangles were used in the experiment (three trials per triangle) that differed in edge length. This was done to ensure that participants needed to carefully attend to the movement along each edge in order to infer how far on the last edge had to be traversed in order to complete the triangle. That is, participants were aware that the triangles in the task may differ in size, but they were not aware of the extent or variety of spatial scale used. In this case, displacement information along each edge of the triangle was critical to estimating the distance to the starting point because, to the participant’s knowledge, triangles may share an identical length of one edge but differ in spatial scale if the other two edges were different. Participants indicated their response using a two button Lumina response pad (Cedrus Corp.) using their index finger if they believed the movement ended at the same point it started and their middle finger to indicate it ending at a different point. Displacement from the starting point was always in relation to total distance traveled rather than angular displacement (i.e., each experimental clip displayed 180° of rotation). This task has been shown to produce a high amount of inter-individual variability (Arnold et al., 2013) which is ideal for investigating the differences in BOLD signal change correlated with performance. Control trials consisted of video clips showing first person movement along a straight line in the same virtual environment in order to control for BOLD signal changes associated with optic flow within the environment that did not have spatial relevance. Experimental and control trials were separated with an interstimulus interval displaying a fixation cross on screen that varied in length between 4 and 6 s.

### MRI ACQUISITION

Participants were scanned using a 3T GE Signa scanner with an 8-channel head coil at the Seaman Family MR Research Centre at the University of Calgary. Stimuli were projected onto a mirror placed in front of the participant’s head within the scanner. Following calibration and shimming, 2D anatomical scans using a T1-weighted fast SPGR sequence where acquired along the AC-PC axis and used to localize the functional scans. Next, task-based fMRI data was acquired using a T2^*^-weighted Echo planar imaging (EPI) sequence (TR = 2500 ms, 45 slices, fov = 24.0 cm, 3 mm × 3 mm × 3 mm voxels in interleaved acquisition, flip angle = 77, 171 volumes) while participants performed the path integration task. Additional orientation tasks were performed while participants were in the scanner, but are not discussed in the present manuscript. The order of each functional run was randomized for each participant to limit any effect of fatigue throughout the scan session. Lastly, high-resolution 3D anatomical images were acquired using a SPGR sequence (fov = 25.6 cm, 1 mm × 1 mm × 1 mm voxels, flip angle = 11, 180 slices).

### MRI DATA PREPROCESSING

All fMRI data was preprocessed using SPM8 (Wellcome Trust Centre for Neuroimaging, Institute of Neurology, UCL; http://www.fil.ion.ucl.ac.uk/) inside MATLAB v.7.14.0.739 (Mathworks, Natick, MA, USA). Six dummy volumes that were acquired prior to task-based volumes to allow for magnetization stabilization were discarded. Each participant’s 3D anatomical scan was first co-registered to the MNI152 ICBM T1 template provided in SPM8. These images were then stripped of the skull and segmented into gray matter (GM), white matter (WM), and cerebral spinal fluid (CSF). Functional images from the path integration task were then realigned to the mean functional image using a six-parameter rigid body transformation to control for motion artifacts. These functional images were then co-registered to each participant’s 3D anatomical image, normalized to the MNI152 template using the transformation matrix generated by SPM8 during anatomical segmentation and resampled into 2 mm voxel space. Lastly, functional images were smoothed with a 8 mm FWHM Gaussian filter.

### MRI DATA ANALYSIS

Preprocessed fMRI data were analyzed using PLS (McIntosh et al., 1996; Krishnan et al., 2011). Two types of PLS were conducted. The first, termed a task PLS, is a multivariate technique that employs singular value decomposition to fMRI data in order to identify LVs. Prior to analysis, activity of each voxel is averaged across each condition block and normalized to the first two volumes (following removal of six dummy volumes to allow for magnetization stabilization) of the run in which participants viewed a fixation cross. The resulting data matrix contains voxel level deviations relative to the grand mean across each condition block. This data matrix is then subjected to singular value decomposition to identify LVs. The LVs identified by a task PLS are composed of distributed spatiotemporal patterns of voxel activity that show similarities within an experimental condition and differences between conditions across the whole group of participants. Each LV contains three vectors. The first vector contains a singular value that represents the strength of the effect for the LV. The second vector contains task saliences that indicate the degree that each task is related to the voxel activity depicted by the LV. The third vector contains voxel saliences, which are numerical voxel weights that identify the collection of voxels most related to the effects express in the LV. Note that for each LV, there is only one salience per voxel that applies for all tasks. In the present study, the task PLS was used to identify the distributed pattern of voxel activity that discriminated BOLD activity in the experimental and control trials of the path integration task.

The second PLS analysis conducted is termed a seed behavior PLS (sbPLS). This analysis examines the pattern of functional connectivity between a seed voxel and the rest of the brain that supports more accurate path integration. Here, our behavior of interest was accuracy on estimating path distance from the experimental trials and the seed was the peak voxel within the PFC identified in the task PLS. The correlation between accuracy and voxel activity of the seed region with all other voxels in the brain was computed across participants during the path integration task, resulting in a matrix containing within-task behavior-seed-brain correlation maps. Singular value decomposition was then carried out to produce three new vectors: singular values, task saliences and the singular image of voxel saliences. As in task PLS, the vector coding singular values indicates the strength of the effect expressed in an LV. Variation in the task salience vector is used to determine whether an LV represents similarity or differences in the behavior-seed-brain correlations across tasks. The voxel saliences indicate the corresponding spatial pattern of activity across the brain. In the present study, the sbPLS was used to identify the spatial patterns of BOLD activity which correlated with BOLD signal from a representative seed voxel in PFC and that also correlated with good and poor performance on estimating path distance. Because sbPLS calculates the covariance between voxel saliences in a given LV, it is a measure of functional connectivity in which the spatial patterns depicted in an LV identify networks that show synchronized activity (McIntosh and Gonzalez-Lima, 1991; McIntosh et al., 1997).

In both task PLS and sbPLS, statistical assessment is calculated through permutation tests for the identified LVs and bootstrap estimation of the standard errors for voxel saliences. Permutation tests are used to test whether the spatiotemporal patterns in each LV are significantly different from random noise (McIntosh et al., 1996) by using sampling without replacement to reassign the order of conditions for each participant. PLS are then calculated for each sample from the permutation tests and the frequency at which the permuted singular values exceed the observed singular values from the original fMRI data are computed. 500 permutation tests were conducted for each analysis. Bootstrap estimations are then conducted on any significant LVs identified during the permutation tests. Bootstrap estimations are designed to test the reliability of non-zero voxel saliences in the LVs using sampling with replacement, where experimental conditions are fixed for all the subjects. This ensures that the patterns expressed in the LVs are stable across participants. For each bootstrap test, PLS is recalculated and voxel saliences displaying subject-specific variability are treated as less reliable. Two hundred bootstrap tests were conducted for each analysis. Bootstrap ratios (BSRs) are proportional to *z*-scores, but are interpreted as a confidence interval with an approximate *p*-value. As PLS calculates the voxel saliences in one mathematical step across the whole brain, it is not necessary to correct for multiple comparisons as it is in traditional univariate analyses on fMRI data. A thorough overview of the application of PLS to block designs is documented in (McIntosh et al., 1996) and visual schematics of the step-by-step process in PLS are available in (Krishnan et al., 2011).

## RESULTS

### PATH INTEGRATION ACCURACY

As predicted from an earlier study (Arnold et al., 2013), participants displayed a high amount of inter-individual variability on estimating path distance (*M* = 3.21, *SD* = 1.42). Performance on the task did not differ between genders, *t*(12) = -1.64, *p* = 0.13, and was not significantly correlated with age, *r* = 0.35, *p* = 0.22.

### NEURAL BASIS OF VISUAL PATH INTEGRATION – TASK PLS

**FIGURE 2:**
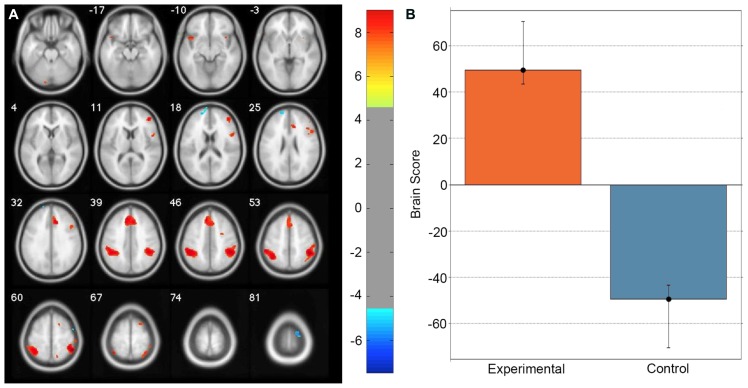
**Singular image for the LV (*p* <0.0001) from the task PLS differentiating path integration and optic flow trials. (A)** Red and yellow regions represent stable increases in BOLD activity across the entire group of participants during path integration trials; blue regions represent stable increases in BOLD activity during control trials. These regions suggest top-down control systems were engaged during experimental trials, which interacted with spatial attention and working memory systems to evaluate metric and angular displacement within the virtual environment. **(B)** Bar graph showing mean brain score for each task condition with its associated confidence interval. Brain score represents the degree to which each task condition was associated with the LV. Displayed in neurological convention (Left = Left).

A task PLS analysis was carried out to identify the brain regions that were engaged by the entire group during all path integration trials regardless of accuracy. The permutation test identified a single LV (*p* < 0.001) that discriminated voxel activity associated with path integration from optic flow. These results are summarized in **Table 1** and **Figure 2**. Dominant positive voxel saliences with a BSR of 4.5 (*p* < 0.0001) indicating brain regions showing significant increases in BOLD signal during the path integration task were found bilaterally in the inferior parietal lobe (IPL) extending into the intraparietal sulcus (IPS), middle (MFG) and superior frontal gyrus (SFG), anterior insula (AI), and precentral gyrus, as well as the right rolandic operculum, precuneus, and the left cerebellum (see **Figure 2A**). These regions suggest the engagement of top-down control systems (Dosenbach et al., 2008; Cole et al., 2013) that interact with spatial attention (Kastner and Ungerleider, 2000; Corbetta and Shulman, 2002; Silk et al., 2010) and working memory (Haxby et al., 2000; Müller and Knight, 2006; Ikkai and Curtis, 2011) systems to initiate attentional control toward the optic flow cues needed to monitor, store and evaluate metric and angular displacement within the virtual environment. Importantly, these results resemble the pattern of BOLD activity identified by Wolbers et al. (2007) in their experimental vs. control trial contrast, verifying that the path integration tasks engaged similar neurocognitive processes across the different groups of participants analyzed in each study. Dominant negative voxel saliences associated with the control task were found in the left superior frontal and middle orbital gyrus, as well as the precentral gyrus in the right hemisphere. Notably, voxel activity was not identified within the HC.

**Table 1:** Dominant positive and negative voxel saliences identified by a single LV (*p* <0.001) in the task PLS differentiating path integration from optic flow trials.

Peak voxel location	*x* (mm)	*y*(mm)	*z* (mm)	BSR	# of voxels
**Positive voxel saliences**
Right middle frontal gyrus	44	44	14	6.5285	181
Left superior medial gyrus	-8	32	42	7.0468	1128
Left middle frontal gyrus	-50	22	36	4.9005	14
Left anterior insula	-44	14	-12	6.0122	126
Right anterior insula	38	12	-10	4.9957	20
Right superior frontal gyrus	20	8	64	5.6855	78
Left precentral gyrus	-44	8	34	4.7632	15
Right rolandic operculum	52	6	14	6.0591	209
Right precentral gyrus	28	-2	46	5.0246	34
Left inferior parietal lobe	-52	-40	52	7.6141	1490
Right inferior parietal lobe	48	-48	58	9.0248	1476
Left cerebellum	-36	-48	-40	5.3209	86
Right precuneus	14	-64	62	4.86	18
Left cerebellum	-14	-88	-22	4.6365	10
**Negative voxel saliences**
Left superior frontal gyrus	-18	58	22	-6.1875	141
Left middle orbital gyrus	-2	48	-6	-4.6494	13
Right precentral gyrus	26	-16	80	-7.4768	98

*Positive voxel saliences represent the spatial pattern of BOLD activity associated with path integration. Peak voxels for each cluster are reported in MNI space.
*

### DIFFERENTIAL FUNCTIONAL NETWORKS ASSOCIATED WITH PATH INTEGRATION ACCURACY – SEED BEHAVIOR PLS

The results of the task PLS indicate the neural activity shared across the entire group during the path integration task. However, we were also interested in whether differential configurations of task-active neural networks were related to accurate performance. To investigate this, a sbPLS was carried out to measure the correlation between accuracy scores on the path integration task and fMRI data collected during each trial of the task. For the seed region, we selected the peak voxel in the PFC (MNI coordinates: *x* = -8, *y* = 32, *z* = 42) in order to investigate whether memory systems were differentially configured between individuals. This region was selected as it showed the most consistent voxel activity during the experimental task in the PFC, a region hypothesized to be critical for spatial working memory systems in both humans (Haxby et al., 2000; Curtis et al., 2005; du Boisgueheneuc et al., 2006) and non-human primates (Qi et al., 2011). As such, it is the most plausible region to show individual differences in spatial memory networks that may be associated with behavioral differences on the task.

**FIGURE 3:**
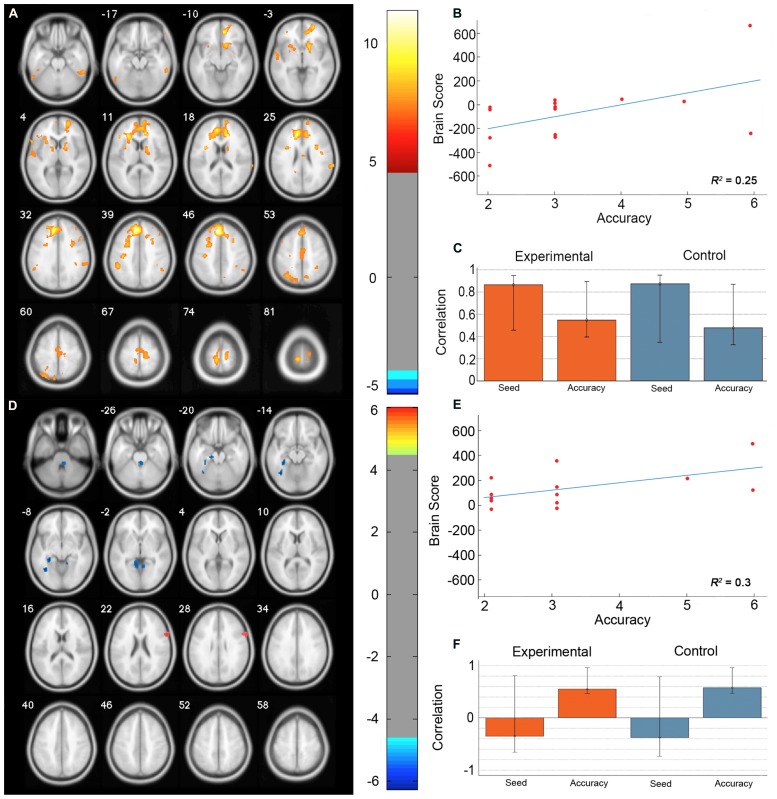
**Results from the seed behavior PLS. (A)** Singular value image from LV1 (*p* < 0.001). Red and yellow regions represent brain areas where increased BOLD activity was associated with high accuracy on the path integration task and correlated with BOLD signal changes in the peak PFC voxel from the task PLS (MNI coordinates *x* = -8, *y* = 32, *z* = 42). This PFC networks suggested increased expression of spatial attention and working memory systems during path integration correlates with more accurate estimation of motion displacement. **(B)** Correlation between individual brain scores from LV1 and accuracy in estimating path distance. **(C)** Correlation between LV1 and data matrix columns representing the seed voxel activity and behavioral accuracy across experimental and control trials. Error bars represent bootstrapped 95% confidence intervals. **(D)** Singular value image from LV2 (*p* < 0.02). Blue regions represent brain areas where increased BOLD activity was associated with low accuracy on the path integration task. This non-PFC network suggests low performance was correlated with the recruitment of a neural system that is typically engaged during landmark navigation and tasks involving allocentric memory. **(E)** Correlation plot showing the relationship between brain scores on LV2 and accuracy in estimating path distance. **(F)** Correlation between LV2 and data matrix columns representing seed voxel activity and behavioral accuracy across experimental and control trials. Error bars represent bootstrapped 95% confidence intervals. Note that error bars cross *r* = 0 for the seed column, indicating that LV2 depicts a functional network that is independent of voxel activity from the PFC seed. Displayed in neurological convention (Left = Left).

The permutation test identified two LVs (LV1: *p* < 0.001; LV2: *p* < 0.02) that showed different functional networks related to performance. These results are summarized in **Table 2** and **Figure 3**. At a BSR of 4.5 (*p* < 0.0001), LV1 identified a functional network that covaried with the PFC seed region and supported better performance (**Figure 3B**). This network included regions bilaterally within the MFG, inferior frontal gyrus (IFG) and supramarginal gyrus, and the superior parietal lobule (SPL), caudate nucleus, anterior cingulate cortex (ACC), angular gyrus, and precuneus in the right hemisphere (**Figure 3A**). Additionally, at a BSR of 4.5 (*p* < 0.0001), LV2 identified a separate functional network that did not covary with the PFC seed region and was found to support lower accuracy performance on the task (**Figure 3E**). This network included areas within bilateral lingual gyrus and cerebellum, and the left posterior parahippocampal gyrus (PHG) and fusiform gyrus (see **Figure 3D**). As with the task PLS, neither LV in the sbPLS included voxel activity within the HC.

**Table 2:** Cluster peaks showing brain regions where increased BOLD activity was associated with path integration accuracy for LV1 (*p* <0.001) and LV2 (*p* < 0.02).

Location	*x* (mm)	*y* (mm)	*z* (mm)	BSR	# of voxels
**LV1**
**Positive voxel saliences**
Left frontal pole	-34	46	0	4.7715	10
Right MFG	32	44	36	4.9309	14
Right MFG	42	40	34	5.4199	53
Left SFG	-8	36	44	11.4555	4944
Right IFG	54	34	2	5.3351	17
Left IFG	-44	30	12	6.4713	45
Left IFG	-60	20	4	6.4415	34
Right caudate nucleus	14	16	-6	7.9933	641
Left central opercular cortex	-32	6	16	8.2881	616
Right IFG	32	4	26	6.6613	394
Left superior temporal gyrus	-56	-2	-2	5.3878	188
Right post central gyrus	46	-6	24	6.1557	35
Right ACC	2	-12	28	4.7444	27
Left post central gyrus	-36	-22	40	6.5316	198
Cerebellum	32	-32	-40	8.3865	325
Left precentral gyrus	-8	-32	78	7.3563	1494
Right supramarginal gyrus	70	-36	22	7.5075	219
Right supramarginal gyrus	40	-40	36	5.3851	49
Left temporal fusiform cortex	-42	-40	-22	5.025	32
Left supramarginal gyrus	-46	-44	40	6.4563	237
Right cerebellum	46	-46	-34	7.489	273
Right SPL	32	-46	52	5.2481	66
Left posterior cingulate gyrus	-14	-50	28	5.1196	10
Right angular gyrus	50	-52	40	5.3019	78
Left inferior temporal gyrus	-54	-56	-20	5.3201	128
Right lateral occipital cortex	20	-58	72	5.0006	12
Left lateral occipital cortex	-32	-66	56	6.1561	521
Right precuneus cortex	10	-66	52	5.1857	76
**LV2**
**Positive voxel saliences**
Right precentral gyrus	64	10	22	6.2696	129
**Negative voxel saliences**
Left PHG	-16	-24	-20	-4.9517	27
Left PHG	-28	-36	-10	-5.9881	93
Cerebellum	-2	-44	-38	-5.5513	292
Right lingual gyrus	12	-46	-6	-4.645	10
Left lingual gyrus	-10	-50	0	-5.5596	117
Right lingual gyrus	8	-54	0	-4.9551	35
Left fusiform gyrus	-38	-62	-12	-6.0396	105

*Positive BSR clusters indicate brain regions within a functional network that supported more accurate performance. Negative BSR clusters indicate brain regions within a functional network that was associated with low performing individuals. Coordinates localized using the Harvard-Oxford cortical and subcortical structural atlas and reported in MNI space.*

## DISCUSSION

Previous work investigating the neural basis of visual path integration in humans emphasized the role of the HC, MFG, and MST underlying individual differences in performance (Wolbers et al., 2007; Diekmann et al., 2009). However, both the central role of the HC in path integration (Alyan and McNaughton, 1999; Sargolini, 2006; Moser et al., 2008; Shrager et al., 2008) and the hypothesis that animals and humans utilize similar neural systems for path integration (Kim et al., 2013) has been brought into question. Here, we investigated the neural mechanisms of visual path integration in humans using a data driven whole brain analysis which is unbiased with respect to both ROIs and experimental contrasts (McIntosh and Lobaugh, 2004). Our results suggest that visual path integration in humans over short distances can operate through top-down control systems that interact with spatial attention and working memory systems. The configuration of these systems varies between individuals, with an increased engagement of a memory system based primarily in the prefrontal and parietal cortex being associated with more accurate path integration. In contrast, low performing individuals were found to additionally engage a functional network based largely in the posterior parahippocampal and lingual gyrus, areas that have traditionally been associated with landmark based navigation. Considered together, these results indicate that the capacity to visually path integrate in humans is contingent upon the differential configuration of attentional and spatial working memory systems within task control networks. These results also support the shifting perspective of the central role of the HC in human visual path integration, suggesting that, in humans, visual path integration may be more reliant on spatial attention and working memory networks based primarily in PFC and IPL than the HC.

The outcome of the task PLS analysis indicates that visual path integration in humans is dependent on top-down control systems that conjunctively interact with spatial attention and working memory systems. Prominent models of task control networks (Dosenbach et al., 2006, 2008; Cocchi et al., 2013; Cole et al., 2013) suggest that regions within PFC including MFC, SFC, and AI interact with IPL to properly allocate the cognitive systems necessary for attending, storing and evaluating environmental stimuli in response to task demands. Visuospatial attention in humans is known to recruit regions within MFG and IPL (Kastner and Ungerleider, 2000; Thiel et al., 2004; de Schotten et al., 2011), as well as topographically mapped areas along IPS (Szczepanski et al., 2010) that assist in applying context-dependent selection of visual stimuli necessary to meet the demands of an experimental task (Corbetta and Shulman, 2002; Jerde et al., 2012). Additionally, spatial working memory has been shown to rely on regional activity in areas of MFC, SFC, and IPS (Haxby et al., 2000; Müller and Knight, 2006; Ikkai and Curtis, 2011), allowing spatial information from visual stimuli to be maintained and integrated over short periods of time (<1 min). In this context, the results of the task PLS broadly suggest that humans are able to use optic flow cues to path integrate through a frontoparietal network that interacts with the spatial attention and working memory processes necessary to compute metric displacement and angular rotation through an environment.

Outside of the neural systems commonly engaged by the whole group during the path integration task, the sbPLS revealed that individuals vary in the configuration of the functional networks recruited during the task. LV1 identified a functional network that covaried with BOLD signal changes from the peak voxel in PFC identified through the task PLS. This network supported high accuracy and was composed of clusters within the right hemisphere in the SPL, angular gyrus, precuneus, anterior caudate nucleus, and ACC, and bilaterally in IFG, MFG, and the supramarginal gyrus. Overall, these regions suggest that increased expression of spatial attention and working memory systems during path integration correlates with more accurate estimation of metric displacement. Regional engagement of SPL, MFG, and IFG have been found in a task-independent network modulating visuospatial attention (Kastner and Ungerleider, 2000; Thiel et al., 2004), while MFG, ACC, and IFG have been associated with tasks requiring spatial working memory (Curtis et al., 2005; Müller and Knight, 2006). We suggest that the functional interactions between spatial working memory regions within the PFC are able to more accurately compute, evaluate and store the optic flow cues provided by movement within the environment using visuospatial attention regulated through parietal regions and egocentric based processing within the precuneus and SPL (Byrne et al., 2007).

Interestingly, BOLD signal change within the right caudate nucleus was found to covary within the network identified by LV1 from the sbPLS. The right caudate nucleus has previously been associated with navigation via a procedural memory system that is independent of HC activity (Hartley et al., 2003; Iaria et al., 2003). However, given the short time frame of experimental trials within the current study, it seems unlikely that participants would recruit a procedural memory system during the path integration task. Rather, inclusion of the caudate nucleus into the functional network may indicate that the spatial computations underlying path integration are utilized by a procedural memory system to track motion through a familiar environment that does not require continuous attention. This information could then be used to signal a mismatch between environmental stimuli and path information coded in procedural memory and used to bring attentional systems back online, consistent with the role of the caudate nucleus in monitoring prediction errors (O’Doherty et al., 2004; Haruno and Kawato, 2006). However, as the present study did not provide explicit feedback, future studies will be needed to more fully delineate the role of the caudate nucleus in path integration and monitoring spatial information relevant to navigation.

In contrast to the functional network identified by LV1 of the sbPLS, LV2 revealed a network associated with lower performance located primarily in the medial occipital-temporal region, including areas within the posterior PHG, lingual, and fusiform gyrus. Considered together, this functional network suggests the recruitment of a neural system that is typically engaged during landmark navigation and tasks involving allocentric memory (Ohnishi et al., 2006; Iaria et al., 2007; Schmidt et al., 2007; Epstein, 2008; Nemmi et al., 2013; Arnold et al., 2014). The regional activity within the left posterior PHG overlapped with the parahippocampal place area (Epstein and Kanwisher, 1998; Epstein et al., 1999), which has been associated with processing visual scenes, particularly environmental features or objects in a scene that help define its spatial context (Mullally and Maguire, 2011). Similarly, the left posterior PHG and lingual gyrus have been found to aid 3D visual transformations of local scenes to spatially locate the position of salient environmental objects (Schmidt et al., 2007). More broadly, activity within the posterior PHG and lingual gyrus has been found in navigation tasks assessing the capacity to form and use allocentric spatial maps of virtual cities (Iaria et al., 2007) and route information based on landmark recognition and their sequential order (Nemmi et al., 2013). In this context, it is plausible to speculate that low performing individuals recruited a neural system that has a primary functional role in spatial navigation for processing, encoding and retrieving the features of visual scenes that form allocentric knowledge of the spatial relationships between landmarks. Additionally, the networks identified by LV1 and LV2 were present in both experimental and control conditions, suggesting that the differential configuration between participants may due to the underlying functional (Deco et al., 2009; Arnold et al., 2014) or structural (van den Heuvel and Mandl, 2009) composition of an individual’s brain.

Comparing the configuration of functional networks engaged by high and low performing individuals, an intriguing possibility is that task control networks differentially recruit spatial memory systems during navigation. In the path integration task, the engagement of spatial working memory systems suited for encoding and retrieving motion displacement based on optic flow cues conferred a behavioral advantage, whereas the recruitment of allocentric memory systems provided little utility given that the virtual environment consisted of only basic textural features with no salient landmarks. This hypothesis is in line with recent research showing that topological properties of allocentric memory networks contribute to behavioral variability in landmark based spatial orientation (Watrous et al., 2013; Arnold et al., 2014). More specifically, it posits that behavioral variability during path integration is related to both the regional involvement of brain areas during task, as well as the broader configuration of task-active neural networks. This hypothesis supports an emerging perspective in the literature that may help explain biases in spatial learning strategies (Wolbers and Hegarty, 2010) through an analysis of how individuals vary in the configuration of the functional networks recruited during navigation. Previous work by (Marchette et al., 2011) have suggested a similar perspective to help understand biases toward place or response learning; however, their observations were not based on analysis of covariance between voxel activates and therefore could not properly delineate functional networks. Future studies investigating topological properties of the structural and functional connections between task control and memory networks across different navigation tasks will be able to directly assess the role of differences in network configuration and their contribution to variability in learning biases.

A notable exception of regional engagement in the current study is the engagement of the HC in the task and sbPLS analyses. While Wolbers et al. (2007) were able to detect differences in HC activity correlating with behavioral measures using an ROI approach, our whole brain analysis did not reveal such results. Our interpretation of this is twofold. First, Wolbers et al. (2007) only investigated fMRI signal obtained during the outward path of the triangle and the response period, whereas the current analysis collapsed across each of the three edges of the triangle. It is possible that HC is only involved initially, perhaps to anchor a starting point within the environment. In such a case, transient activity in the HC may not be identified by our analysis that averaged fMRI signal across the entire task, despite the increased statistical power that PLS affords over mass univariate analyses. However, in the current experimental set up, spatial information about each triangle edge was necessary to accurately locate the starting point, as the triangles varied in spatial scale, possibly minimizing the importance of coding the starting location through a HC mediated mechanism. A second interpretation relates to the behavioral measures. Wolbers et al. (2007) did not find HC activity in the main experimental vs. control task contrast, rather a five voxel cluster within the right HC associated with within subject trial-to-trial variability and a larger 23 voxel cluster in the right HC that correlated with random error in reorienting toward the starting point. In comparison, the present study investigated individual differences in estimating the distance of a homing vector. As such, it may be that HC engagement pertains to accuracy in angular displacement and not distance estimation. Future studies measuring both important components of path integration ability within the same group of individuals may be able to further clarify this discrepancy.

While the lack of HC involvement in task-active functional networks in both high and low performers is in line with recent neuropsychological evidence that path integration ability may be preserved in patients that have undergone partial or complete HC resection (Kim et al., 2013), it is in contrast to previous studies reporting deficits in path integration in similar patients. Two studies in particular found that right, but not left, HC resection patients showed behavioral deficits on path integration tasks assessing linear distance estimates (Philbeck et al., 2004) and turning accuracy (Worsley et al., 2001). However, a critical difference between these studies and the present study is the use of visual information to provide movement cues during path integration. Both neuropsychological studies showing path integration deficits used tasks in with patients were blindfolded, except Philbeck et al. (2004), which included an additional task that did not require blindfolding, but did limit visual information using the spatial layout of the testing environment. As such, it may be that visual path integration is less reliant on the HC in humans and is able to be subserved by the proposed network outlined above. A critical prediction from this is that HC resection patients may be able to visually path integrate during short time scales and perform well on tasks similar to the one utilized here. Future research will be able to directly test this prediction.

As the current study is exploratory in nature, there are several limitations that should be clarified. First, path integration in the real world operates through conjunctive processing of visual, vestibular and proprioceptive signals (Loomis et al., 1999). Due to limitations of fMRI studies, we were only able to directly assess the neural mechanisms supporting visual path integration. However, previous research has shown that path integration ability with optic flow cues alone closely resembles path integration ability with visual, vestibular and proprioceptive information (Kearns et al., 2002) suggesting that visual path integration is a sufficient approximation for how it operates in the external world. Second, although PLS allows for a data driven method to interrogate the spatial pattern of voxel activity that discriminates different task states, with the present design we were unable to investigate how regional activity relates to specific points in time within experimental trials. While our perspective is that behavioral variability in path integration relates to dynamic interactions between various neural networks throughout the brain, future studies may be able to further delineate the functional roles of localized brain regions in computing the spatial signals necessary for path integration using traditional univariate analyses coupled with an understanding of the topological configuration of the functional networks involved. Importantly, it currently remains unclear the precise role that spatial working memory systems may play in path integration. Future research may be able to more fully articulate the role of different regions within the frontal and parietal cortex that show variable activity in relation to modulations of spatial aspects of similar path integration tasks (e.g., spatial scale, degree of angular rotation, total path distance). Third, the current analysis has a limited number of trials from which to draw meaningful patterns of BOLD signal, in particular compared to Wolbers et al. (2007), who analyzed 40 experimental trials. However, in both the task and sbPLS analyses, the number of trials was sufficient to identify significant LVs that discriminated experimental and control trials, and revealed differential functional networks correlated with accuracy in estimating motion displacement. This suggests that the neural patterns of activity are particularly robust in our group of participants, given the high significance of BSRs that the data were interpreted with. Fourth, due to the use of a dichotomous response, the group distribution was similar to what might be expected from random guessing. However, based on three main points we believe participant’s were effortfully responding during the task: (a) high performing individuals (accuracy > 3) performed better than chance, (b) previous work in a larger sample (*n* > 200) showed similar participants perform better than chance on the task (Arnold et al., 2013), and (c) anecdotal reports from the participants during debriefing that they were engaged in effortful responding throughout the task. Lastly, due to our experimental paradigm, we are unable to properly disentangle task control, attention, and memory systems across the task and whether they can be functionally dissociated from one another. The dynamic cooperation and competition of functional networks during task states represents a promising new line of research (Cocchi et al., 2013) that may be able to more fully articulate the precise dynamic relations of the neural systems underlying human path integration.

In sum, the results of the present study extend upon previous attempts to characterize the neural mechanisms in human path integration (Wolbers et al., 2007). Our data driven analysis did not identify task activity or behavioral variability associated with HC engagement, lending support to the perspective that different brain regions may be more critical for path integration on short time-scales (Alyan and McNaughton, 1999; Moser et al., 2008; Shrager et al., 2008). Rather, our results suggest that humans engage a neural system that consists of task control, spatial attention and working memory regions working in conjunction during path integration. This system is differentially configured between individuals, such that different memory networks relevant to spatial navigation are recruited during path integration. The compatibility of the memory network recruited with the type of sensory signals that can be derived from environmental stimuli allows for either effective or ineffective navigation. These findings support the recent suggestion that humans are able to path integrate using a non-hippocampal spatial working memory system over short distances (Kim et al., 2013), and raises the possibility that different configurations of neural networks may subserve path integration in humans and animals.

## Conflict of Interest Statement

The authors declare that the research was conducted in the absence of any commercial or financial relationships that could be construed as a potential conflict of interest.
